# Predicting the Biodegradation of Magnesium Alloy Implants: Modeling, Parameter Identification, and Validation

**DOI:** 10.3390/bioengineering5040105

**Published:** 2018-11-29

**Authors:** Amirhesam Amerinatanzi, Reza Mehrabi, Hamdy Ibrahim, Amir Dehghan, Narges Shayesteh Moghaddam, Mohammad Elahinia

**Affiliations:** 1 Dynamic and Smart Systems Laboratory, Mechanical Industrial and Manufacturing Engineering Department, The University of Toledo, Toledo, OH 43606, USA; amirhesam.amerinatanzi@utoledo.edu (A.A.); reza.mehrabinejad@gmail.com (R.M.); 2 Mechanical Engineering Department, The University of Tennessee at Chattanooga, Chattanooga, TN 37403, USA; hamdy-ibrahim@utc.edu; 3 School of Mechanical, Industrial and Manufacturing Engineering, Oregon State University, Corvallis, OR 97331, USA; dehghana@oregonstate.edu; 4 Mechanical & Aerospace Engineering, University of Texas at Arlington, Arlington, TX 76019, USA; narges.shayesteh@uta.edu

**Keywords:** biodegradable alloys, magnesium alloy, finite element modeling, corrosion model

## Abstract

Magnesium (Mg) and its alloys can degrade gradually up to complete dissolution in the physiological environment. This property makes these biomaterials appealing for different biomedical applications, such as bone implants. In order to qualify Mg and its alloys for bone implant applications, there is a need to precisely model their degradation (corrosion) behavior in the physiological environment. Therefore, the primary objective develop a model that can be used to predict the corrosion behavior of Mg-based alloys in vitro, while capturing the effect of pitting corrosion. To this end, a customized FORTRAN user material subroutine (or VUMAT) that is compatible with the finite element (FE) solver Abaqus/Explicit (Dassault Systèmes, Waltham, MA, USA) was developed. Using the developed subroutine, a continuum damage mechanism (CDM) FE model was developed to phenomenologically estimate the corrosion rate of a biocompatible Mg–Zn–Ca alloy. In addition, the mass loss immersion test was conducted to measure mass loss over time by submerging Mg–Zn–Ca coupons in a glass reactor filled with simulated body fluid (SBF) solution at pH 7.4 and 37 °C. Then, response surface methodology (RSM) was applied to calibrate the corrosion FE model parameters (i.e., Gamma (γ), Psi (ψ), Beta (β), and kinetic parameter (K_u_)). The optimum values for γ, ψ, β and K_u_ were found to be 2.74898, 2.60477, 5.1, and 0.1005, respectively. Finally, given the good fit between FE predictions and experimental data, it was concluded that the numerical framework precisely captures the effect of corrosion on the mass loss over time.

## 1. Introduction

Magnesium (Mg)-based alloys are attractive for bone implant applications, as they corrode gradually in vivo with an appropriate host response, and then degrade completely after the healing of the bone tissue [1,2,3]. These alloys can be designed to degrade within a desired period of time through alloying elements and coating techniques, thus allowing for the regeneration of the surrounding soft or hard tissues [4,5]. When compared with other metallic materials, Mg-based alloys, either crystalline or amorphous, do not significantly interfere with magnetic resonance imaging (MRI), hence allowing for the accurate assessment of the device function and surgical outcome to be made after the surgery [6]. Crystalline Mg-based alloys offer a relatively high mechanical strength (~135–285 MPa) that is suitable in load-bearing applications. On the other hand, amorphous Mg-based alloys have disordered atomic (glass-like) structure. Amorphous Mg-based alloys have been studied for bone fixations applications due to their superior strength and corrosion resistance in comparison with traditional crystalline Mg-based alloys [2,7]. As an instance, the amorphous Mg–Zn–Ca-based alloys showed a range of tensile strength from 675 MPa to 894 MPa [7]. In addition, the adverse effect of stress shielding is prevented, as the amorphous Mg alloys enjoy Young’s modulus values (45 GPa) close to that of the human bone [8,9,10]. Moreover, these alloys have close to bone density (~1.7–2.0 g/cm^3^), while conventional titanium-based alloys, such as Ti–6Al–4V, have much higher density (~4.42 g/cm^3^) when compared to that of natural bone (1.8–2.1 g/cm^3^) [11,12]. Magnesium is naturally present in the human body (~25 g in total, 50–60% of which exist in the bone) [13]. Approximately 380–850 mg of Mg is provided daily via different intake sources such as grains, nuts, and green leaves/vegetables. Most importantly, while Mg-based alloys provide the unique property of enhancing the cell attachment and proliferation through generating magnesium-containing calcium phosphate, , they are considered biocompatible in vivo alloys that can receive acceptable responses from the host environment [14,15,16].

As stated previously, magnesium alloys are interesting because they can degrade over time and leave the human body after the needed period of functionality. However, it is important to understand and control the degradation process of the implemented bioparts. The most common means of assessing the degradation can be performing experiments and doing numerical simulations to have a better insight into their degradation behavior. In general, the numerical modeling of biodegradation is of great interest, since it enables the analysis of structures and performance, which cannot be evaluated in vivo or in vitro. It also reduces the time and cost required for manufacturing large numbers of prototypes [17,18,19]. Finite element (FE) continuum damage mechanics (CDM) models are the most favorable tools for predicting the performance of complex biodegradable geometries, as they are more sensitive to mesh resolution [20]. These models evaluate the corrosion properties of components by means of arbitrary state variables without referring to an explicit description of microscopic phenomena and their evolution. Here, at the end of each time increment, a portion of elements could be removed by FE deletion [21,22,23]. Since a CDM-based FE model is generally phenomenological-based, rather than physical/chemical-based, it does not capture physical/chemical processes related to the factors such as electrochemical surface reactions, species evolution, or species diffusion. Therefore, for each material, the model has to be recalibrated based on the corresponding experimental data [24].

In the literature, only a few research groups have focused on the field of phenomenological modeling of biodegradable alloys through FE modeling based on CDM approaches. Grogan et al. [20] developed a corrosion model to predict the corrosion behavior of AZ31, which is a biodegradable Mg alloy, using pitting corrosion $d_{P}$ models (i.e., a non-uniform breaking down of the sample). In their research, they have considered the calibration of three parameters ($K_{u}$, γ, and β) based on the experimental data (mass loss after a certain periods of times up to 72 h) obtained from the immersion test. They have used “tuning technique” to find these unknown pitting parameters and reported 0.00042, 0.2, and 0.8 for $K_{u}$, γ, and β, respectively. Gastaldi et al. [25] also developed a phenomenological CDM-based FE model to simulate the biodegradation properties of ZK60, ZM21, AZ31, AZ61, and AZ80. Unlike the study done by Grogan et al. [20], they captured the effect of uniform corrosion $d_{u}$ instead of pitting corrosion $d_{P}$, as they observed almost linear behavior in immersion testing for all five alloys for different periods of times up to 100 h. Therefore, they only have reported $K_{u}$ parameter for the alloys, which ranged from 10^−2^ to 10^−1^. Specifically, for AZ31 Mg-based alloy, the value of $K_{u}$ was reported to be 0.00500. However, it is believed that a uniform model for predicting corrosion in immersion testing is not precise compared to a pitting corrosion model [20]. Later, Oppeel et al. [26] also captured the effect of pitting corrosion $d_{P}$ using CDM-based FE approaches to develop their model for AZ31 Mg-based alloy. After modeling the immersion test results and the subsequent tuning, they reported the values of $K_{u}$, γ, and β as 0.00650, 0.2, and 0.5, respectively. 

The major challenge associated with the developed corrosion models in literature is that they have used arbitrary tuning techniques to report their model parameters, which is time-consuming, imprecise, and unrepeatable. In addition, they have neglected the shape factors parameter Psi (ψ) and only considered parameters $K_{u}$, γ, and β. To close this gap, we first developed a customized FORTRAN user material subroutine (VUMAT) code based on CDM FE approaches to include the effect of the pitting corrosion $D_{p}$ of Mg–Zn–Ca alloy. In this study, we considered all main four parameters (i.e., γ, ψ, β, and $K_{u}$) for the calibration of the model rather than only three. Finally, we implemented response surface methodology (RMS), rather than an arbitrary tuning technique, to tune and calibrate the four effective parameters.

## 2. Materials and Methods

### 2.1. Sample Preparation and In Vitro Immersion Corrosion Testing

**Figure 1:**
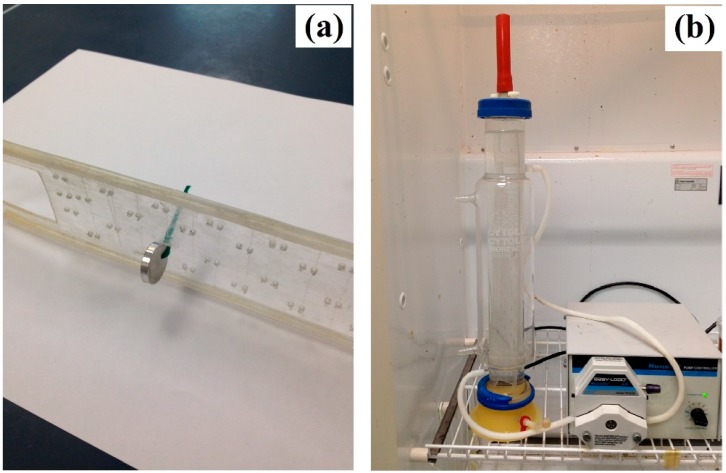
The setup for the glass reactor and the peristaltic pump.

An Mg–1.2Zn–0.5Ca (wt.%) alloy was produced by casting using pure Mg, pure Zn, and a 15 wt.% Ca–Mg master alloy. This alloy composition was found to result in an optimized mechanical and corrosion properties based on our previous studies [27,28]. The melting process was conducted in a steel crucible at 720 °C for 15 minutes under a CO_2_ + 0.5% SF_6_ atmosphere. Then, the melt was cast into a steel permanent mold to create as-cast cylindrical ingots of 16-mm diameter by 100-mm length. Finally, seven samples (coupons of 15-mm diameter and three-mm thickness) were machined from the created ingots for the purpose of immersion testing. The machined samples were then polished using SiC papers (600–2000 grit) and cleaned using ethanol. The polished and ethanol-degreased samples were then connected to a plastic holder from one end, and then submerged in a simulated body fluid (SBF) solution at a monitored pH of 7.4 and a fixed temperature of 37 °C (Figure 1). The composition of the SBF solution is presented in Table 1.

As presented in Equations (1)–(3), the corrosion reaction of Mg in an aqueous environment (physiological environment) produces Mg ions, hydroxyl group (OH), and hydrogen ($H_{2}$). The OH quickly reacts with Mg ions to create a layer of magnesium hydroxide $\mathrm{Mg}{\left(\mathrm{OH}\right)}_{2}$ on the surface of the sample. $\mathrm{Mg}{\left(\mathrm{OH}\right)}_{2}$ may convert into soluble magnesium chloride (${\mathrm{MgCl}}_{2}$). The overall reaction consumes $H^{+}$ and produces ${\mathrm{OH}}^{-}$ from the medium, leading to an increase in the pH value [15,29,30,31].

(1) $$\mathrm{Mg}+2H_{2}O={\mathrm{Mg}}^{2+}+2{\left(\mathrm{OH}\right)}^{-}+H_{2}$$

(2) $${\mathrm{Mg}}^{2+}+2{\left(\mathrm{OH}\right)}^{-}=\mathrm{Mg}{\left(\mathrm{OH}\right)}_{2}$$

(3) $$\mathrm{Mg}{\left(\mathrm{OH}\right)}_{2}+{\mathrm{Cl}}^{-}={\mathrm{MgCl}}_{2}+2\left(\mathrm{OH}\right)$$

To maintain the pH between 7.3–7.8 throughout the experiments, a diluted hydrochloric acid (5M-HCL) was titrated into the SBF every eight hours, and the SBF solution was replenished every two days. The level of temperature was also maintained constant (at 37 °C) by placing the samples in an incubator throughout the period of the tests. The immersion tests were performed on seven coupons, and for each, the mass loss was recorded for five intervals; 0 days, seven days, 14 days, 21 days, and 28 days. For each interval, the mass of the seven samples were recorded and averaged after rinsing the corrosion products on the surface of the samples using a mixture of ${\mathrm{CrO}}_{3}$ (20%) and ${\mathrm{AgNO}}_{3}$ (1%) (Chromic acid) and ethanol [32]. Then, the mass loss (mg/cm^2^) related to each interval was calculated using Equation (4):

(4) $$\text{Mass Loss}=\frac{m_{i}-m_{f}}{A}$$

where mass loss is in (mg/cm^2^), $m_{i}$ is the initial mass of sample before immersion test (mg), $m_{f}$ is the final mass of each sample after immersion test (mg), and A is the sample’s surface area exposed to the SBF solution (cm^2^).

It should be pointed out that $H_{2}$ pockets are formed during corrosion in the rate of one mL for every one mg of Mg, as reported in Equation (1). Hence, measuring the mass loss is enough to simulate the corrosion rate of the Mg samples in addition to being a representation of the amount of $H_{2}$ released.

### 2.2. Damage Model Development

A FORTRAN user material subroutine or VUMAT was developed to investigate the degradation behavior of biodegradable alloys in the FE solver Abaqus/Explicit (Dassault Systèmes, Waltham, MA, USA). Abaqus/Explicit has an interface that allows the user to implement the constitutive relationships of any given arbitrary complexity in addition to the already existed material models in the Abaqus material library. An FE model was used for the phenomenological investigation of the influence of pitting damage on the mechanical behavior of Mg-based alloys. In this study, the corrosion model was developed based on CDM theory, which was proposed by Lemaitre et al. [22], through the introduction of a scalar damage parameter $d_{P}$ to assess the overall damage as corrosion proceeds. According to Wenman et al. [24], the values of $d_{P}$ range from zero to one, corresponding to the intact and fully corroded material element. Therefore, the elements with $d_{P}$ = one should be eliminated from the FE mesh, simulating the mass loss of the corroded alloys. However, Abaqus does not allow elements to be deleted. In order to solve this issue, the mechanical properties of corroded elements were set to nearly zero, and for virtual representation, the corroded elements were set to be invisible.

The following steps and equations are utilized in order to apply the defined model to the investigations. In Equation (5), $K_{u}$($h^{-1}$) is the kinetic parameter representing the uniform corrosion process, $\delta_{u}$ is the material characteristic, $L_{e}$ (mm) is the FE model characteristic length, and $\lambda_{p}$ is an element-specific dimensionless pitting parameter, all of which are initially assigned to each element:

(5) $$\frac{\partial d_{p}}{\partial t}=\frac{\delta_{u}}{L_{e}}\;K_{u}\;\lambda_{p}$$

To assign $\lambda_{p}$ values to all elements on the initial exposed surface, a probability density function (PDF) f(x) of a Weibull random variable was used (Equation (6)). γ and ψ are dimensionless distribution shape factors characterizing the probability density function (PDF). The probability of the value of $\lambda_{p}$ ranging from “a” to “b” is given by Equation (7). In Abaqus, when an element is effectively removed, the neighboring elements ($\lambda_{p}^{\prime }$) inherit the value of the pitting parameter $\lambda_{p}$ of the completely corroded and eliminated elements. β is the dimensionless scaling parameter controlling pitting growth acceleration. Equation (8) explains how the pitting parameter is assigned to neighboring elements.

(6) $$f\left(x:\;\psi ,\;\gamma \right)=\{\begin{array}{llll} \frac{\gamma }{\psi }{\left(\frac{x}{\psi }\right)}^{\gamma -1}e^{-{\left(x/\psi \right)}^{\gamma }} & & & x\geq 0\\ 0 & & & x<0 \end{array}$$

(7) $$P\;\left[a\leq \lambda_{p}\leq b\right]=\int_{a}^{b}f\left(x\right)\mathrm{dx}$$

(8) $$\lambda_{P}^{\prime }=\beta \;\lambda_{P}$$

**Figure 2:**
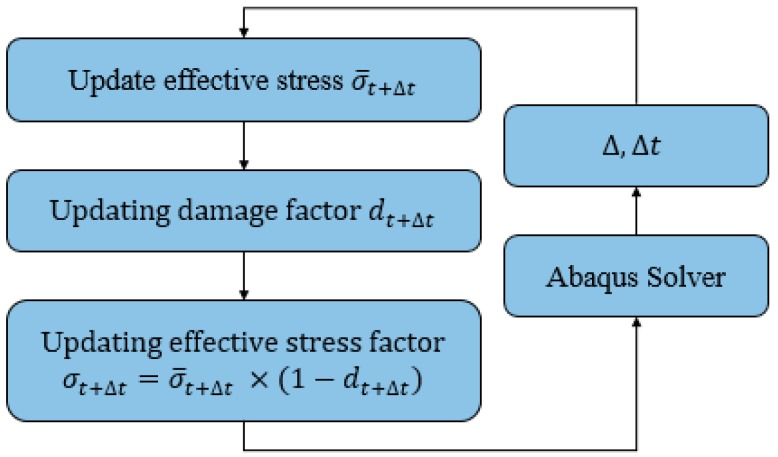
Schematic overview of the steps toward generating the VUMAT code.

**Figure 3:**
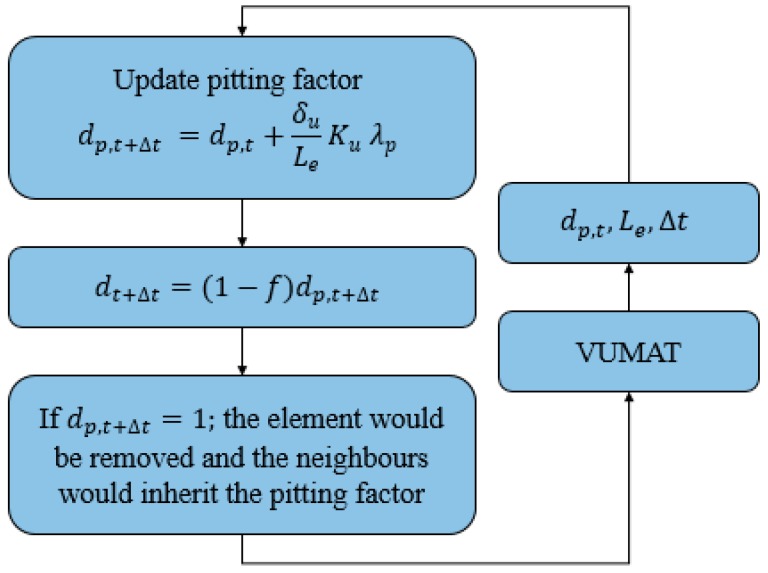
Schematic overview of the subroutine.

The flowchart presented in Figure 2 and Figure 3 demonstrate the implementation of this user-defined material model, as well as the details related to each step. 

### 2.3. Boundary Conditions and Simulations

Coupons that were 15 mm in diameter and three mm in thickness were modeled using SOLIDWORKS (Dassault Systèmes, Waltham, MA, USA), and then were imported to Abaqus/Explicit for FE Analysis. Next, the developed VUMAT, which was compatible with the FE solver Abaqus/Explicit, was implemented into the FE model to simulate the corrosion behavior of the Mg–1.2Zn–0.5Ca (wt.%) alloy. The coupons were meshed in Hypermesh (Hyperworks, Troy, MI, USA) with an eight-node linear brick (C3D8), which represents three-dimensional (3D), solid, hexagonal, and deformable element types. In addition, mesh convergence studies were conducted to guarantee a minimal influence of the mesh on the simulation results. In order to reproduce the mass loss versus time period curves, corrosion degradation process was evaluated for different periods of time (0 days, three days, seven days, 14 days, 21 days, and 28 days).

### 2.4. Calibration Strategy through RSM

The aim of this section is to propose a method to calibrate the four effective corrosion parameters to results in an acceptable match between the mass loss–time curves predicted by the model and the observed ones from the immersion experiments presented in Section 2.1. The common calibrating approach in the literature for the corrosion parameters is based on the trial and error techniques. In such approaches, a set of corrosion parameters are initially considered, and the FE results are compared with those of the experimental works. The parameters are then changed, and the modeling data are regenerated to the point that the FE results are in accordance with the experimental findings. Although the outcomes of these techniques seem to be close enough, time consumption, cost inefficiency, non-repeatability, and lack of needed accuracy for the intended applications reduce their applications. In this research, the root mean square error (RMSE) technique is implemented to calibrate the corrosion parameters. MiniTab v16 was used to generate the design of experiment (DOE) matrix and analyze the response surface models. In this technique, a three-level, four-factor Box–Behnken design was selected, because it was capable of evaluating the quadratic interactions between pairs of corrosion parameters while minimizing the number of required experiments (cheaper, more accurate, and time-efficient) [33,34]. 

Here, experimental data (mass loss versus time) of immersion tests for the fabricated Mg–1.2Zn–0.5Ca alloy were used. The effective parameters of the immersion tests were as following: Gamma (γ), Psi (ψ), Betha (β), and Kinetic ($K_{u}$). Using the RSME technique, the impact of parameters and their interactions were evaluated. The selected ranges for these factors are presented in Table 2. To define the discrepancy between the experimental and modeling results, the Chi-square value ($\chi^{2}$) was calculated for each run based on Equation (9). A total number of 27 combinations of effective corrosion parameters were considered, and $\chi^{2}$ was measured for each experiment. These combinations are indicated for the Mg–1.2Zn–0.5Ca alloy, as listed in Table 3.

(9) $$\begin{array}{l} \chi^{2}=\frac{{\left(\mathrm{observed}-\mathrm{predicted}\right)}^{2}}{\mathrm{observed}}\\ \;=a_{1}\gamma +a_{2}\psi +a_{3}\beta +a_{4}U_{k}+a_{5}\gamma^{2}+a_{6}\psi^{2}+a_{7}\beta^{2}+a_{8}U_{k}^{2}+a_{9}\gamma \psi \\ \;+a_{10}\gamma \beta +a_{11}{\gamma U}_{k}+a_{12}\psi \beta +a_{13}{\psi U}_{k}+a_{14}{\beta U}_{k}+a_{15} \end{array}$$

## 3. Results

### 3.1. Degradation Behavior of Mg–Zn–Ca Alloy 

**Figure 4:**
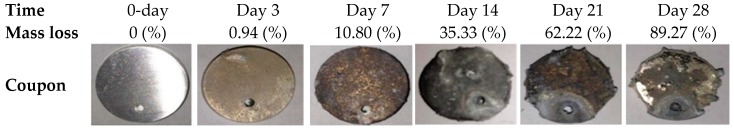
Mass loss of Mg–1.2Zn–0.5Ca (%) coupon as a function of time during immersion testing in SBF at pH 7.3–7.8 and 37 °C for a period of 28 days.

Figure 4 shows the mass loss of the Mg–1.2Zn–0.5Ca (wt.%) coupon obtained from immersion test in corrosive environment (SBF solution) at a pH of 7.3-7.8 and temperature of 37 °C for a period of 28 days. The in vitro mass loss of the coupon was recorded to be 0.94%, 10.80%, 35.33%, 62.22% and 89.27 after three days, seven days, 14 days, 21 days, and 28 days, respectively. 

### 3.2. Mesh Analysis

**Figure 5:**
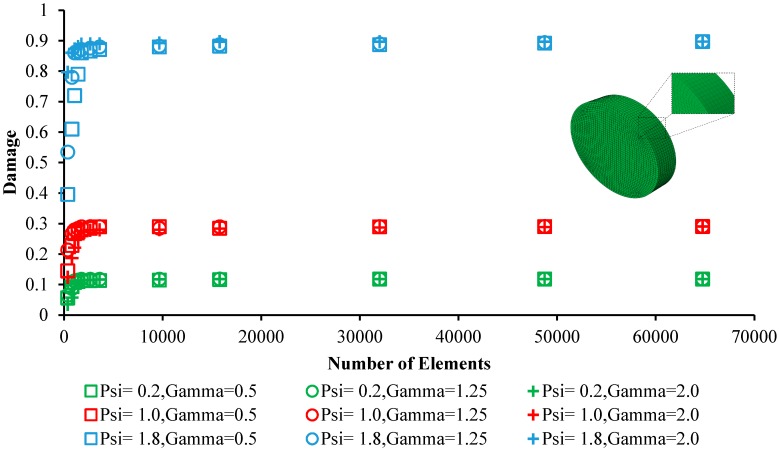
Mesh convergence analysis of the corroding Mg–1.2Zn–0.5Ca (wt.%) coupon in corrosive environment (SBF solution) at 37 °C. The meshed coupon is also presented in the figure.

Figure 5 shows the mesh convergence studies as well as the virtual representation of the meshed coupon for the simulation of immersion testing in corrosive environment (SBF solution) for the Mg–1.2Zn–0.5Ca (wt.%) alloy. The FE mesh is also shown in the outset. The eight-node linear brick elements (C3D8) was used to mesh the coupons to provide higher element metrics (quality) and better modal analysis. Mesh convergence study was conducted to evaluate the sensitivity of the resultant FE damage factor to the element size, as well as other effective parameters (i.e., γ, ψ). To this aim, a single coupon was meshed using 12 different element numbers, ranging from 365 to 64750 elements, as well as nine different combinations between ψ (range from 0.2 to 1.8) and γ (range from 0.5 to 2). Each of the 108 models was run using Abaqus, and the resultant damage factor was recorded after a constant time. An aspect ratio of close to one was considered for all of the elements, as the most suitable value to model degradation-induced mass loss [26]. As it is seen, a mesh leading to errors less than 5% was acceptable while it resulted in a comparable damage factor to that of utilizing a very fine mesh, i.e., 64,750 elements. The final mesh was composed of 3600 elements.

### 3.3. Determination of Model Parameters 

**Figure 6:**
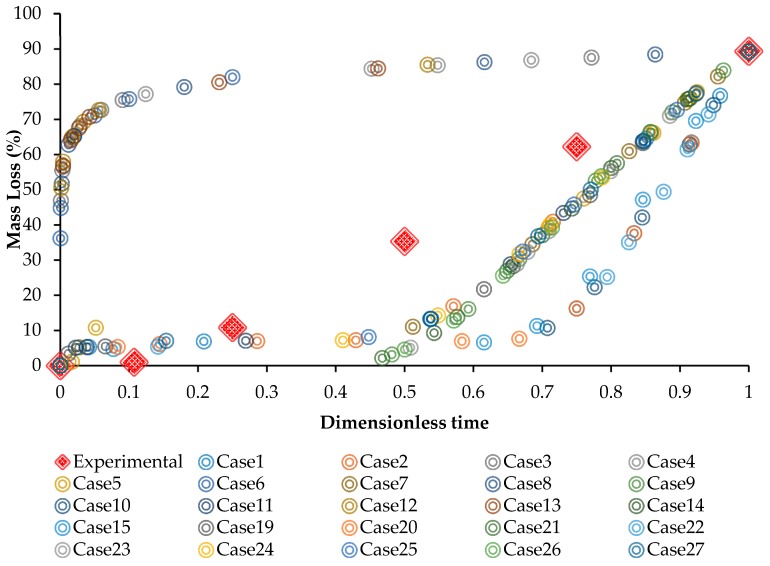
The mass loss (%) vs. dimensionless time obtained from the immersion experiment of Mg–1.2Zn–0.5Ca as well as the 27 finite element (FE) model proposed by the RSM technique.

Table 4 represents the 27 combinations between the effective pitting corrosion parameters ($\gamma ,\;\psi ,\;\beta ,\;K_{u}$) defined by Minitab v16 software. The results of immersion experiment (see Section 3.1) were also used as a reference for calibration of these parameters. Next, 27 different runs in Abaqus were conducted until the acquired mass loss exceeded 89.27%, which was the maximum mass loss of the experiment recorded after 28 days. Then, for each condition, the $\chi^{2}$ was calculated using Equation (9). Finally, the set of pitting parameters that resulted in the lowest $\chi^{2}$ = 0.034 was proposed as following: (γ=2.74898, ψ=2.60477, β=5.1, and $K_{u}=0.1005$). This set of pitting parameters was defined as the model parameters for simulating the corrosion behavior of Mg–1.2Zn–0.5Ca (wt.%) in immersion testing. For a better clarification, the mass loss versus dimensionless time are plotted in Figure 6 for the 27 different conditions as well as the immersion experiment. 

**Figure 7:**
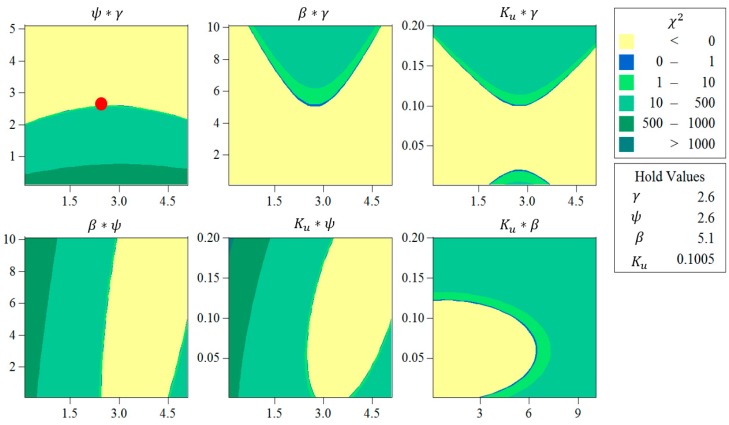
Contour plots representing the distribution of $\chi^{2}$ as a function of the pitting parameters (γ, ψ, β, and $K_{u}$).

Figure 7 also demonstrates the contour plots, representing the distribution of $\chi^{2}$, as the result of using different sets of pitting parameters. As it is clear in the figure, each of the pitting parameters (i.e., γ, ψ, β, and $K_{u}$) has a significant effect (i.e., p value less than 0.05) on the resultant mass loss. Here, the regions in blue color are preferable compared to those in green, as they are attributed to lower $\chi^{2}$ values, and therefore, there is a lower discrepancy between the experimental results and FE modeling. The selected optimum values for the effective parameters (γ = 2.74898, ψ = 2.60477, β = 5.1, and $K_{u}$ = 0.1005) are also shown as a red circle in Figure 7.

### 3.4. Evaluation of the Model 

**Figure 8:**
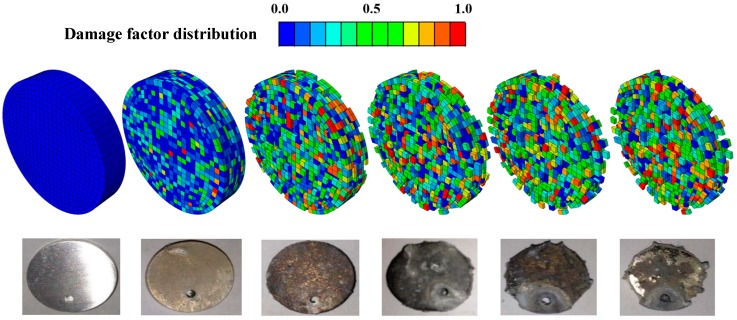
FE experimental results for immersion testing of Mg–1.2Zn–0.5Ca. The color code represents the value of the damage parameter d ranging from 0 to 1.

**Figure 9:**
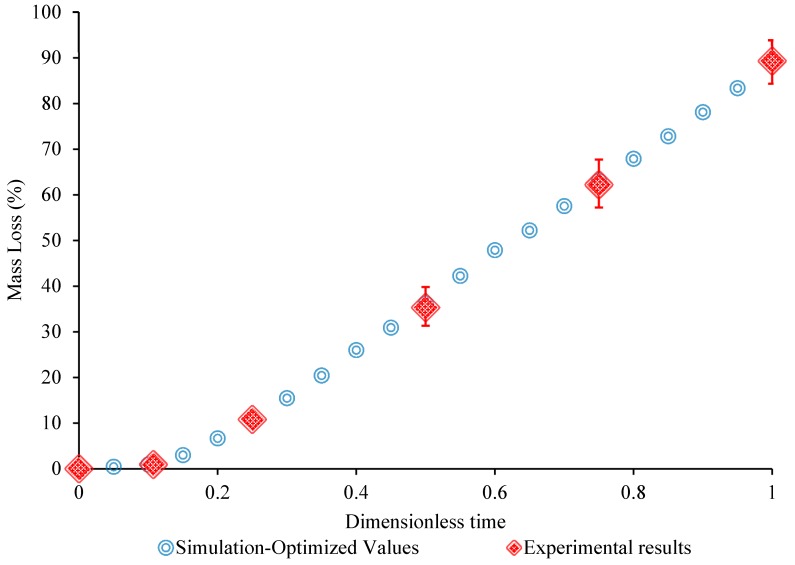
Mass loss (%) vs. dimensionless time as measured in immersion test of Mg–1.2Zn–0.5Ca alloy (n = 7) and corresponding FE simulation predictions.

Figure 8 shows the evolution of the damage parameter throughout the Mg–1.2Zn–0.5Ca (wt.%) coupon volume as a function of corrosion time (0 days, three days, seven days, 14 days, 21 days, and 28 days), while the suggested parameters in Section 3.3 are used in the model. It can be observed that the pitting-like material is simulated by means of the developed numerical framework. In Figure 9, the FE results using the optimum parameters are also compared to the experimental results obtained from immersion testing for the alloy. It is clear that the use of optimum pitting parameters in the developed model accurately reproduce the obtained experimental results ($\chi^{2}$ = 0.034; *p* value = 0.012).

Table 5 demonstrates a collection of reported model parameters by the research groups in the literature (Grogan et al. [20], Gastaldi et al. [25], and Oppeel et al. [26]), as well as the parameters presented in this work.

## 4. Discussion

In this study, a phenomenological corrosion model based on the CDM approach has been developed to simulate the degradation of Mg-based alloys, including Mg–1.2Zn–0.5Ca (wt.%). Hence, the simulation is based on the overall macroscopic behavior of the alloy rather than taking into account the micro and/or nanoscale physical mechanisms that govern degradation. Therefore, calibrating the effective degradation constant parameters for each particular biodegradable alloy is needed. Due to the phenomenological basis of the developed model, it does not physically capture the effect of the electrochemical processes on the corrosion behavior, meaning that its predictions are specific to a given alloy. 

As presented in Table 5, a significant discrepancy is observed between the FE modeling predictions in literature and the corresponding experimental data (the calculated $\chi^{2}$ was at least 25.14, which was reported by Oppeel et al. [26]). However, the presented model in this work demonstrated a negligible discrepancy between the modeling and experimental results (the calculated $\chi^{2}$ was 0.034).

**Figure 10:**
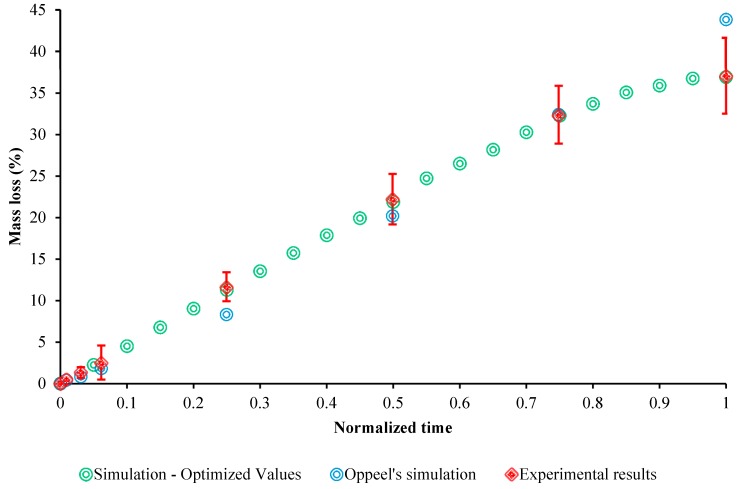
Experimental (n = 3) and FE representation of the AZ31 alloy using the developed model versus Oppeel et al [26]. (Immersion testing).

To evaluate the time efficiency and accuracy of our model compared to the one by Oppeel et al. [26], the proposed model was recalibrated in order to acquire the pitting parameters for three AZ31 components using their immersion testing data. A similar approach as explained in Section 2.3 was implemented to calibrate the four effective parameters for AZ31 alloy (note: In their work, the mass loss was collected from immersion testing on five samples after a period of 96 h). Finally, the set of pitting parameters that resulted in the lowest $\chi^{2}$ = 0.0399 was proposed as following: (γ=0.5846, ψ=0.93003, β=0.2505, and $K_{u}=0.005$). However, the calculated $\chi^{2}$ for the immersion prediction by Oppeel et al. [26] was about 25.14. This means that the proposed corrosion model closely matches the observed results in experiment. Figure 10 shows the modeling results of our model versus their modeling and experimental data. 

In summary, this model enables the prediction of the in vitro corrosion behavior of degradable alloys (crystalline and amorphous) through recalibrating the four effective parameters using the RSM approach. These four parameters include β (the dimensionless scaling parameter controlling pitting growth acceleration), γ (the dimensionless distribution shape factors characterizing the probability density function (PDF)), ψ (the dimensionless distribution shape factors characterizing the probability density function (PDF)) and $K_{u}$ (the kinetic parameter representing the uniform corrosion process). The values of mass loss over time have to be collected for the purpose of obtaining these parameters. It should be pointed out that the model that was developed in this study has a number of limitations. Due to its phenomenological basis, it is required to recalibrate the effective degradation parameters for each particular biodegradable alloy. Also, as the model predictions are not generated based on the alloy microstructure, hence, the model cannot be used in predicting the effects of precipitate formation or grain size on corrosion. In addition, the model does not capture the effects of tissue coverage on alloy corrosion if implanted inside the body. 

## 5. Conclusions

This work focuses on the development and calibration of a model capable of precisely predicting the corrosion behavior of Mg-based alloys using the RSM approach. To calibrate the pitting parameters ($\gamma ,\;\psi ,\;\beta ,\;K_{u}$), an immersion experiment was also performed on Mg–Zn–Ca coupons. The results predicted by the developed model demonstrated a close matching between the modeling and experimental results of (RMSE = 2.8 × 10^−6^, *p* value = 0.001). Therefore, the presented method enables us to predict the corrosion behavior of Mg-based alloys more accurately with a reduction in cost and time of calculations.

## Figures and Tables

**Table 1:** Amounts of reagents for the preparation of 1000 mL solution of the simulated body fluid (SBF), adopted from Oyane et al. [29].

Reagent	Amount
NaCl	5.403 g
NaHCO_3_	0.504 g
Na_2_CO_3_	0.426 g
KCl	0.225 g
K_2_HPO_4_·3H_2_O	0.23 g
MgCl_2_·6H_2_O	0.311 g
0.2 mol L^−1^ NaOH	100 mL
HEPES	17.892 g
CaCl_2_	0.293 g
Na_2_SO_4_	0.072 g
1 mol L^−1^ NaOH	15 mL

**Table 2:** The ranges of effective pitting parameters: (Mg–1.2Zn–0.5Ca—Immersion test).

Variables, Unit	Range and Levels		
	−α	0	α
γ	0.1	2.6	5.1
ψ	0.1	2.6	5.1
β	0.1	5.1	10.1
$K_{u}$	0.001	0.1005	0.2

**Table 3:** The 27 suggested sets of pitting parameters obtained from the response surface methodology (RSM) technique to be able to predict the Chi-square value ($\chi^{2}$) for each experiment: (Mg–1.2Zn–0.5Ca—immersion test).

Case	(γ)	(ψ)	(β)	K_u_
**1**	2.6	5.1	5.1	0.001
**2**	2.6	2.6	5.1	0.1005
**3**	2.6	0.1	5.1	0.2
**4**	0.1	0.1	5.1	0.1005
**5**	5.1	2.6	5.1	0.2
**6**	2.6	0.1	5.1	0.001
**7**	0.1	2.6	5.1	0.2
**8**	5.1	0.1	5.1	0.1005
**9**	2.6	2.6	0.1	0.001
**10**	2.6	5.1	10.1	0.1005
**11**	2.6	2.6	10.1	0.001
**12**	2.6	0.1	10.1	0.1005
**13**	2.6	2.6	5.1	0.1005
**14**	0.1	2.6	0.1	0.1005
**15**	0.1	5.1	5.1	0.1005
**16**	2.6	2.6	5.1	0.1005
**17**	0.1	2.6	5.1	0.001
**18**	2.6	0.1	0.1	0.1005
**19**	5.1	2.6	5.1	0.001
**20**	2.6	5.1	5.1	0.2
**21**	5.1	2.6	0.1	0.1005
**22**	5.1	5.1	5.1	0.1005
**23**	2.6	2.6	0.1	0.2
**24**	5.1	2.6	10.1	0.1005
**25**	0.1	2.6	10.1	0.1005
**26**	2.6	5.1	0.1	0.1005
**27**	2.6	2.6	10.1	0.2

**Table 4:** Obtained $\chi^{2}$ for 27 different set of pitting parameters obtained from the RSM technique. The optimized pitting parameters are provided based on the lowest value observed for $\chi^{2}$.

Case	(γ)	(ψ)	(β)	K_u_	$\chi^{2}$
**1**	2.6	5.1	5.1	0.001	1.42
**2**	2.6	2.6	5.1	0.1005	0.43
**3**	2.6	0.1	5.1	0.2	1409.05
**4**	0.1	0.1	5.1	0.1005	342.21
**5**	5.1	2.6	5.1	0.2	6.31
**6**	2.6	0.1	5.1	0.001	703.62
**7**	0.1	2.6	5.1	0.2	0.96
**8**	5.1	0.1	5.1	0.1005	460.68
**9**	2.6	2.6	0.1	0.001	0.59
**10**	2.6	5.1	10.1	0.1005	1.52
**11**	2.6	2.6	10.1	0.001	1.16
**12**	2.6	0.1	10.1	0.1005	1096.31
**13**	2.6	2.6	5.1	0.1005	0.43
**14**	0.1	2.6	0.1	0.1005	0.42
**15**	0.1	5.1	5.1	0.1005	1.67
**16**	2.6	2.6	5.1	0.1005	0.43
**17**	0.1	2.6	5.1	0.001	0.36
**18**	2.6	0.1	0.1	0.1005	634.60
**19**	5.1	2.6	5.1	0.001	6.41
**20**	2.6	5.1	5.1	0.2	1.71
**21**	5.1	2.6	0.1	0.1005	0.68
**22**	5.1	5.1	5.1	0.1005	0.59
**23**	2.6	2.6	0.1	0.2	0.56
**24**	5.1	2.6	10.1	0.1005	0.47
**25**	0.1	2.6	10.1	0.1005	0.47
**26**	2.6	5.1	0.1	0.1005	0.70
**27**	2.6	2.6	10.1	0.2	0.32
**Optimized**	**2.74898**	**2.60477**	**5.1**	**0.1005**	**0.034**

**Table 5:** The calibrated parameters by different groups in the literature. Gamma (γ), Psi (ψ), Beta (β), and kinetic parameter ($K_{u})$.

Parameter	Grogan et al. [20]	Gastaldi et al. [25]	Oppeel et al. [26]	Our Simulation	
**Material**	AZ31	AZ31	AZ31	Mg-Zn-Ca	AZ31
γ **(−)**	0.2	-	0.2	2.74898	0.5846
ψ **(−)**	-	-	-	2.60477	0.93003
β **(−)**	0.8	-	0.5	5.1	0.2505
**K_u_ (h^−1^)**	0.00042	0.00500	0.00650	0.1005	0.005
$\chi^{2}$	34.83	48.73	25.14	0.034	0.0399
